# A Novel Design of Complementary Split Ring Resonator Metamaterial-Based Low-Profile MIMO Antenna with Defected Ground Structure for S/C/X/Ka Band Applications

**DOI:** 10.3390/mi14061232

**Published:** 2023-06-11

**Authors:** Meshari Alsharari, Sunil Lavadiya, Khaled Aliqab, Ammar Armghan, Malek G. Daher, Shobhit K. Patel

**Affiliations:** 1 Department of Electrical Engineering, College of Engineering, Jouf University, Sakaka 72388, Saudi Arabia; 2 Department of Information and Communication Technology, Marwadi University, Rajkot 360003, India; 3 Physics Department, Islamic University of Gaza, Gaza P.O. Box 108, Palestine; 4 Department of Computer Engineering, Marwadi University, Rajkot 360003, India

**Keywords:** MIMO antenna, complementary split ring resonator, metamaterial, DG, gain, patch antenna

## Abstract

The article represents the design of two port-based printed MIMO antenna structures that have the advantages of low profile, simple structure, good isolation, peak gain, directive gain, and reflection coefficient. The performance characteristics are observed for the four design structures by cropping the patch region, loading the slits near the hexagonal-shaped patch, and adding and removing the slots in the ground area. The antenna provides a least reflection coefficient of −39.44 dB, a maximum electric field of patch region of 33.3 V/cm, a total gain of 5.23 dB, and good values of total active reflection coefficient and diversity gain. The proposed design provides nine bands’ response, a peak bandwidth of 2.54 GHz, and a peak bandwidth of 26.127 dB. The four proposed structures are fabricated using a low-profile material to support mass production. The comparison among simulated and fabricated structures is included to check the authenticity of the work. The performance assessment of the proposed design with other published articles is carried out for the performance observation. The suggested technique is analyzed over the wideband of frequency region 1 GHz to 14 GHz. The multiple band responses make the proposed work suitable for wireless applications in S/C/X/Ka bands.

## 1. Introduction

The use of wireless communication technology and the ongoing quest for small portable devices have undergone remarkable development during the last decade. There is potential for new wireless systems to enable a wide variety of applications and numerous users simultaneously. However, the need for an enhanced data rate is severely hampered by the limited bandwidths available due to spectral congestion. Therefore, technologies that may be used in wireless devices to perform antenna operating at several frequency bands are in high demand [1].

The ability to send data rapidly gives it a lot of leeway when it comes to using the spectrum, which might be helpful for mobile computing devices in the future. The broad bandwidth and rapid data transfer speeds are ideal for various applications such as short-range radars, surveillance devices, and medical imaging [2,3,4]. Wideband technology, which attempts to give high data rates and acceptable prices, is possible. There are several advantages to using a wideband system. It can transmit small information pulses at high data rates with lower power levels and less radio interference than a narrowband system [5]. The actual deployment of 5G systems depends on the efficient design of 5G antennas [6], which are fundamental components of any communication system. The gain and efficiency of mm-wave 5G antennas and their bandwidth are expected to be high. The benefits of printed antennas, such as their flat construction and design simplicity, have led to significant studies in mm-wave wireless communications.

In addition, the metamaterial property helps to achieve better antenna performance. For MIMO technology to function, it is essential that the antenna components used by the transmitter and receiver be independent of one another. In order to measure the performance of an antenna array in MIMO systems, numerous metrics have been proposed in [7].

The miniaturization of the MIMO structure has been shown to reduce the isolation between antenna components in [8]. The decoupling technology in MIMO using 3D metamaterial structures improves antenna isolation. In [9], the reduction in mutual coupling using defective isolation is presented. Article [10] suggests using an isolated radial resonator for enhancing isolation among two elements of a patch in the MIMO antenna. A novel technique to reduce interference amongst the many antennas used in 5G’s proposed MIMO design is suggested in [11]. The deployment of 5G networks relies on the development of an effective antenna structure for 5G networks. The great spectral efficiency and low power consumption of massive MIMO have made it a frontrunner for the 5G network.

In [12], the antenna represents the lower isolation, and bandwidth is important in MIMO antenna design. Article [13] shows a broad bandwidth and high gain as being the most significant. The dipole antenna equipped with a metamaterial array provides a high gain in the end-fire direction. The article demonstrates a change in the center frequency due to shared ground and very poor isolation between the antenna’s components. Article [14] represents a greater level of interfering noise hinders signal transmission due to −10 dB isolation between antenna band notch characteristics for two-port MIMO, and an antenna is proposed in [15], with a lower gain of 2 dBi. Article [16] presents a method for achieving a wider bandwidth of 2.1 to 11 GHz. Two quasi-self-complimentary MIMO antennas have been suggested in [17] to attain an ultra-wide bandwidth. In addition to being small, the antenna provides a significant gain. While the envelope correlation coefficient (ECC) and isolation are lower in quasi structure, the arrangement of the structure in a better way enables less isolation and ECC. Diversity constraints are not explored for the suggested quasi-shaped antenna. The four-element-based mushroom-shaped design provides up to −40 dB of isolation over a frequency range of 2.3–2.4 GHz. The complex design and vast size are partly due to the mushroom’s multi-layered structure. The broadband four-elements-based MIMO antenna is presented in [18,19]. The inverted L-monopole structure is used to attain the broad bandwidth. Nevertheless, only −11 dB of isolation can be accomplished throughout the operational bandwidth. The partial diffracted ground and diagonally trancing patch help to attain sub-6 GHz communication applications [20]. The insertion of stub in the resonators helps with improving isolation [21]. The transparent MIMO antenna using transparent conductive oxide and plexiglass substrate is presented in article [22]. The machine-learning-enhanced MIMO antenna allows for more precise aiming across a wider variety of use cases [23]. The current trend uses graphene-based THz MIMO antennas; however, fabrication technology needs to be explored further.

There is a need for high-gain, low-cost, multiband antenna to be designed, which can operate over a wide range with multiple bands. This manuscript presents the 1 × 2 MIMO antenna, which is analyzed for the L, S, C, and X frequency bands. The main objective of the proposed work is to achieve the multiband response with healthy gain and sufficient wideband response to target multiple applications for S, C, X, and Ka bands. The presented manuscript represents the novel concept of the addition of complementary split rings and slots in the patch area and the ground region. The modification in the patch structure and ground structure helps to achieve the targeted goals. The presented work shows the path to achieving a broad bandwidth, lower E.C.C., and good gain using the low-profile substrate material, which helps to achieve satellite communication applications. 

## 2. Design of MIMO Antenna

**Figure 1:**
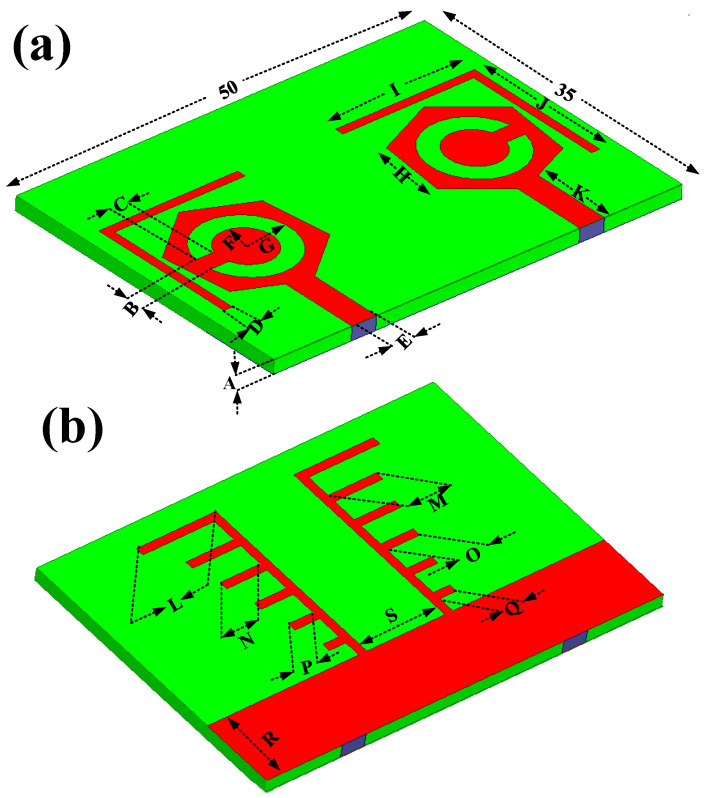
The three-dimensional view of the proposed antenna. (**a**) 3D top view. (**b**) 3D lower view of the proposed structure.

Four designs were considered to analyze the reflection/transmission coefficient: peak gain, directivity, electric fields, and radiation patterns. Figure 1 characterizes the three-dimensional view of the top side and bottom side. All of the dimensions were considered in the millimeter. The overall dimensions of the design were 50 × 35 mm^2^. The lumped port was applied to the structure for the excitation. The patch and ground layers were designed by considering them as copper material. The thickness of the patch and ground layers was 0.35 mm. The substrate height was 1.6 mm. The substrate material was chosen to be FR-4 epoxy, as it is inexpensive and easy to fabricate. FR4 has dielectric constant of 4.4 [24]. The novelties of the structure are mentioned below.

- The proposed MIMO antenna design incorporates CSRR metamaterial, which offers unique electromagnetic properties, such as negative permeability and permittivity. This integration enables the antenna to exhibit enhanced performance characteristics, including improved bandwidth and radiation efficiency.
- The antenna design incorporates a defected ground structure to mitigate surface wave propagation and improve the isolation between antenna elements.
- The proposed MIMO antenna design achieves a low profile and compact form factor, making it suitable for various space-constrained applications. In addition, the antenna’s reduced size and planar structure contribute to its versatility and ease of integration into modern wireless communication devices.
- The antenna design offers wideband operation across the S/C/X/Ka frequency bands, covering various communication applications. In addition, the CSRR metamaterial and DGS implementation contribute to the achieved broad operating bandwidths, ensuring compatibility with multiple frequency bands.
- A sufficient reflection coefficient, more than 1 GHz of bandwidth, and a healthy gain are achieved for all of the proposed designs.
- Design and fabrication were carried out using FR4 dielectric material to check the reliability of the structure. The FR4-based low-profile material was used to achieve targeted goals, which helped with cost reduction.
- The four design structures were analyzed based on modifying the DGS and slit around the patch region to check the performance of different forms.
- The proposed design is compared with the other existing methods to identify the improvement in the structure.
- The simulated and measured results are compared to judge tolerance among them. Lower tolerance among both results is observed.
- Overall, the proposed antenna design exhibits excellent characteristics for MIMO applications. It demonstrates low mutual coupling between antenna elements, high isolation, good radiation efficiency, healthy gain, and proper diversity parameters. These attributes contribute to enhanced channel capacity, increased data rates, and improved system performance in MIMO systems.

The different antenna structures were examined by inserting CSRR in the patch with slits outside of the patch and defective ground structure. The insertion of a CSRR in a patch antenna can have several effects on the performance of a MIMO antenna, as follows: The CSRR can alter the resonant frequency of the patch antenna. Due to a unique metamaterial property, it is possible to shift the resonant frequency of the patch antenna to a desired frequency. This adjustment can be beneficial in achieving better frequency selectivity and matching with other antennas in the MIMO system. The CSRR can widen the bandwidth. By introducing the CSRR, the effective electrical length of the antenna structure can be modified, resulting in a broader frequency bandwidth. This expanded bandwidth can be advantageous for MIMO systems that require simultaneous operation over multiple frequency bands. In MIMO antenna systems, mutual coupling between the individual antennas can degrade performance by interfering with signal transmission and reception. The CSRR can help to reduce mutual coupling between the antennas by creating isolation between them. It can act as a decoupling element, decreasing the coupling effects and improving the isolation between the antennas. The insertion of a CSRR can alter the radiation pattern of the patch antenna. The CSRR can affect the current distribution and electric field distribution on the patch, leading to changes in the radiation characteristics. Properly designed CSRR structures can be used to shape the radiation pattern and improve the antenna’s performance in terms of gain, directivity, and radiation efficiency. MIMO systems often benefit from polarization diversity, where different antennas are designed to transmit and receive signals with different polarizations. The CSRR can help to enhance the polarization diversity of the patch antenna by modifying its polarization properties. By carefully designing the CSRR, it is possible to achieve improved polarization isolation and better performance in terms of cross-polarization discrimination.

The incorporation of a defective ground structure (DGS) in a antenna can have several effects on its performance, as follows: Cross-polarization refers to the undesired reception or transmission of signals with a polarization orthogonal to the desired polarization. DGSs can help to suppress cross-polarization in MIMO antennas. By introducing appropriate DGS patterns or slots in the ground plane, the radiation characteristics and polarization properties of the antenna can be controlled, leading to improved polarization isolation and reduced cross-polarization. DGSs can be used to shape and control the radiation pattern of MIMO antennas. By incorporating specific DGSs, such as slots or patches, in the ground plane, the current distribution and electromagnetic field distribution on the antenna can be manipulated. This enables control over the antenna’s radiation pattern, beam steering, and directivity, which can be advantageous in MIMO systems to optimize coverage, increase gain, or mitigate interference. DGSs can help to mitigate interference from external sources or neighboring antennas by introducing appropriate DGSs.

Using structures in the ground plane, such as slots or fractal patterns, it is possible to suppress the effects of nearby interfering signals or electromagnetic noise, improving the signal quality and overall performance of the MIMO system.

The antenna design technique is analyzed in four essential parts in order to explain the working concept of the planned MIMO antenna. Table 1 shows the four structures of the proposed design. DS-1 consists of two hexagonal-shaped MIMO structures along with one circular-shaped portion engraved in the patch area. Two slits are also placed on the outer side of the hexagonal-shaped patch region. Two arrays of lines and one plane region are available in the diffracted ground region.

DS-2 has two hexagonal-shaped MIMO structures without a circular shape. Two slits are also placed on the outer side of the hexagonal-shaped patch region. Two arrays of lines and one plane region are available in the diffracted ground region. DS-3 has two hexagonal-shaped MIMO structures with a circular-shaped portion engraved in the patch area. There are not any slits in the outer place of the hexagonal-shaped patch region. Two arrays of lines and one plane region are available in the diffracted ground region. DS-4 has two hexagonal-shaped MIMO structures with one circular-shaped portion engraved in the patch area. In addition, there are slots available in the outer place of the hexagonal-shaped patch region. The ground region is simple without array lines. The DGS is a technique used in the design of antennas and R.F. circuits to reduce electromagnetic coupling and improve isolation between antenna elements in MIMO systems. The DGS in the ground plane helps in reducing coupling in MIMO antennas through the following mechanisms:

- Electromagnetic isolation: In a MIMO system, each antenna element should ideally radiate and receive signals independently without interfering with each other. However, due to the close proximity of multiple antennas, there can be coupling between them, resulting in interference and the degradation of system performance. By introducing DGSs in the ground plane, the coupling between the antenna elements can be minimized. The DGS creates an electromagnetic barrier between the antennas, reducing the mutual coupling and enhancing isolation.
- Surface wave suppression: DGSs can also suppress surface waves propagating on the ground plane. Surface waves can lead to increased coupling between antennas and reduce isolation. By incorporating DGSs in the ground plane, the surface waves can be attenuated, reducing their impact on the antenna elements and minimizing the coupling between them.
- Impedance matching: DGSs can be designed to provide additional degrees of freedom in tuning the impedance of the antenna elements. By appropriately designing the shape and size of DGSs, the impedance seen by each antenna element can be adjusted. This helps in achieving a better impedance match between the antenna elements, minimizing reflections and optimizing the overall system performance.
- Enhanced radiation efficiency: The presence of DGSs in the ground plane can also improve the radiation efficiency of the antenna elements. By reducing the coupling and surface wave effects, the energy that would otherwise be lost in coupling or surface wave propagation is efficiently radiated by the antennas. This leads to improved overall system performance and better signal quality.

**Figure 2:**
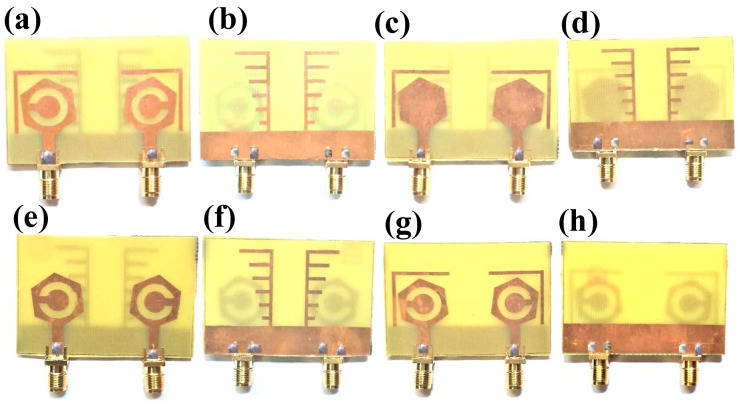
Fabricated proposed structures. (**a**,**b**) Proposed design structure—1. (**c**,**d**) Proposed design structure—2. (**e**,**f**) Proposed design structure—3. (**g**,**h**) Proposed design structure—4.

The fabrication of the proposed structures is carried out for analysis purposes, and indifferent proposed structures are shown in Figure 2. The dimensions of different regions are presented in Table 2.

The circular-shaped ring is cut in the hexagonal-shaped patch area. The width *W* of the patch is calculated using Equation (1) [25].

(1) $$W=\frac{C_{0}}{2fr}\sqrt{\frac{2}{\varepsilon r+1}}$$

where *C*_0_ is the light speed, *W* is the patch width, fr is the resonating frequency, and εr is the dielectric constant of the substrate. Fringing radio waves travel between patch and ground via air and substrate. Due to the difference in the dielectric constants of substrate and air, the practical dielectric constant calculation is required. The effective dielectric constant (Ɛ_𝑒𝑓𝑓_) is determined using Equation (2).

(2) $${Ɛ}_{eff}=\frac{\varepsilon r+1}{2}+\frac{\varepsilon r-1}{2}{\left[1+12\frac{h}{w}\right]}^{-0.5}$$

The fringing effect changes the effective area of the patch. Therefore, variation in the area is represented by Δ*L*. The change is calculated using the following Equation (3).

(3) $$\frac{\Delta L}{h}=0.412\frac{(\varepsilon eff+0.3)\left(\frac{w}{h}+0.264\right)}{(\varepsilon eff-0.258)\left(\frac{w}{h}+0.8\right)}$$

where *h* is the substrate’s height. The exact dimension of the patch can be calculated using Equation (4).

(4) $$L=\frac{1}{2fr\sqrt{\varepsilon eff\mu_{0}\varepsilon_{0}}}-2\Delta L$$

## 3. Result Analysis and Discussion

**Figure 3:**
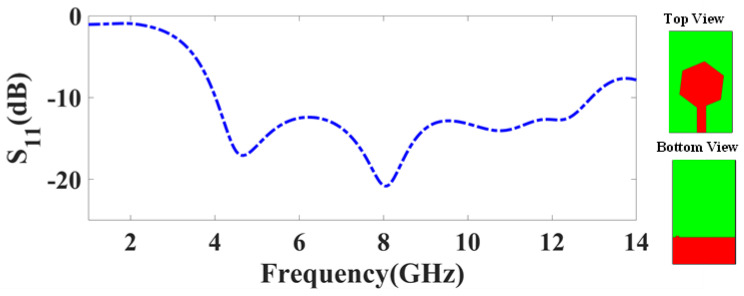
Proposed one-element MIMO antenna.

Each design was numerically analyzed using the full-wave higher-frequency structural simulator (HFSS). A variety of performance metrics such as return loss, radiation pattern, total gain plot, field distribution, gain for the different frequencies, directivity, TARC, and directivity gain were analyzed. The reflection and transmittance response must be analyzed for the high-frequency antenna structure [26]. The initial analysis was carried out for the one-element MIMO antenna to check the performance observation. The analysis in terms of reflection coefficients for the one-element MIMO structure is represented in Figure 3. There are two bands with less than −10 dB of reflection coefficients observed. The first band is shown at 4.5 GHz with an S_11_ of −18 dB and the second band is shown at 8 GHz with an S_11_ of −21 dB.

**Figure 4:**
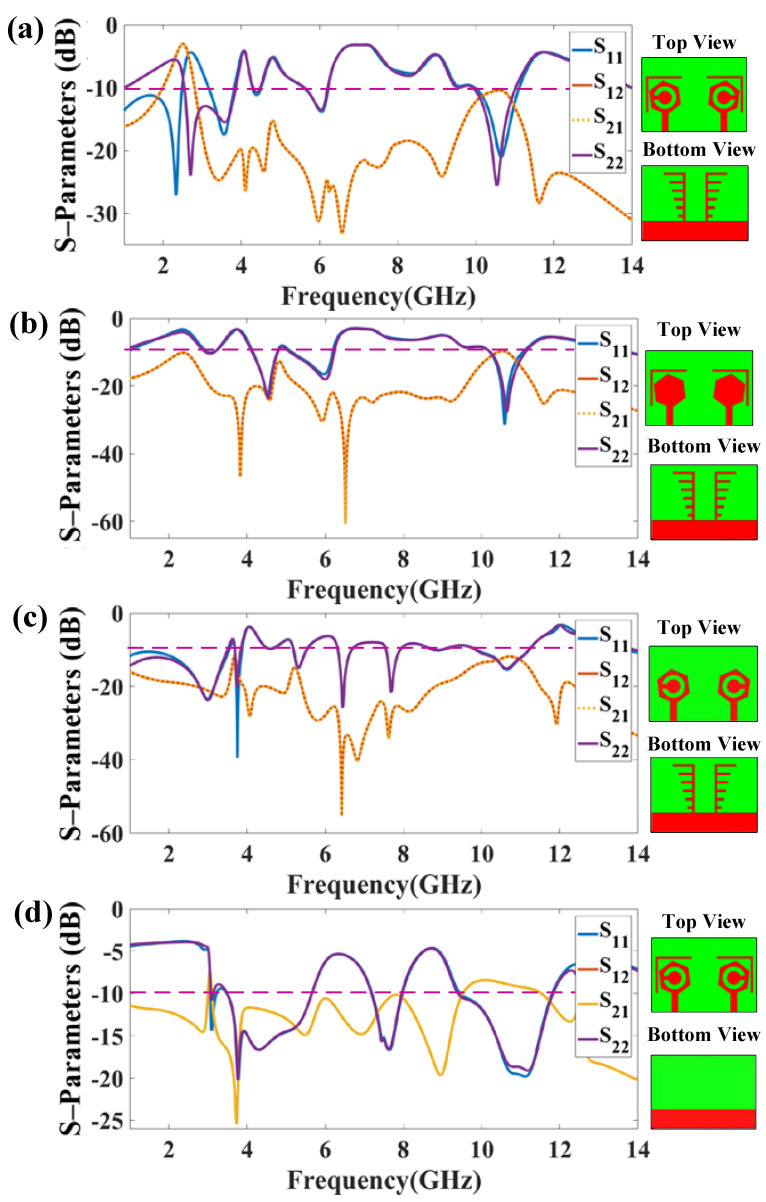
Reflection and transmission coefficients of the proposed four structures. (**a**) DS-1 provides the two frequency band responses, The design provides peak isolation of −27.098 dB and maximum bandwidth of 2.485 dB. (**b**) DS-2 provides the five band responses with peak isolation of −31.457 dB and maximum bandwidth of 1.045 dB. (**c**) DS-3 provides the nine band responses with peak isolation of −39.44 dB and maximum bandwidth of 2.54 dB. (**d**) DS-4 provides the five band responses with peak isolation of −20.308 dB and maximum bandwidth of 2.35 dB. The maximum bandwidth of 2.485 GHz was attained for the DS-1 configuration. There were a maximum of nine bands of reflection response attained for the DS-3 configurations, and the maximum isolation was attained for the DS-3 design. The comparison of the measured and simulated reflection plots is presented in Figure 5. The measured results authenticate the performance results of both structures. The rationale of the proposed design was analyzed based on the modification of the patch and defective ground region.

The MIMO structure’s unusual form primarily aids in attaining higher isolation (<−28 dB) during measurement as per DS-1. Two bands of reflection response are observed in Figure 4. The first band represents reflection coefficients of −27.098 dB at 2.3 GHz, and the S_11_ is less than −10 dB in the range of 1 to 2.485 GHz. The second band provides the S_11_ of −17.507 dB at 10.5 GHz; this band is observed in the range of 3.165 GHz to 3.825 GHz. Therefore, a peak bandwidth of 2.485 GHz is attained in this configuration. 

DS-2 shows the five bands with less than −10 dB value. The first band represents reflection coefficients of −10.16 dB at 3 GHz; this band is observed in the range of 3.005 GHz to 3.14 GHz. The second band provides an S_11_ of −23.172 dB at 4.5 GHz; this band is observed in the range of 4.15 GHz to 4.775 GHz. The third band is shown in the range of 5.145 GHz to 6.19 GHz with an S_11_ of −16.572 dB at 5.5 GHz. The fourth band is shown in the range of 10.215 GHz to 11.05 GHz with an S_11_ of −31.457 dB at 10.5 GHz. Finally, the fifth band is shown in the range of 13.76 GHz to 14 GHz with an S_11_ of −10.69 dB at 13.9 GHz. In this mode, a maximum bandwidth of 1.045 GHz is achieved.

DS-3 shows the eight bands of reflection coefficients with less than −10 dB value. The first band represents reflection coefficients of −23.425 dB at 3 GHz; this band is observed in the range of 1 GHz to 3.54 GHz. The second band represents reflection coefficients of −39.44 dB at 3.8 GHz; this band is observed in the range of 3.69 GHz to 3.865 GHz. The third band represents reflection coefficients of −15.075 dB at 5.2 GHz; this band is observed in the range of 5.19 GHz to 5.52 GHz. The fourth band represents reflection coefficients of −25.319 dB at 6.5 GHz; this band is observed in the range of 6.325 GHz to 6.675 GHz. The fifth band represents reflection coefficients of −21.620 dB at 7.8 GHz; this band is observed in the range of 7.555 GHz to 7.915 GHz. The sixth band represents reflection coefficients of −10.147 dB at 8.9 GHz; this band is observed in the range of 8.765 GHz to 8.985 GHz. The seventh band represents reflection coefficients of −15.075 dB at 5.3 GHz; this band is observed in the range of 5.19 GHz to 5.52 GHz. The eighth band represents reflection coefficients of −15.497 dB at 10 GHz; this band is observed in the range of 9.86 GHz to 11.215 GHz. The ninth band represents reflection coefficients of −10.759 dB at 13.8 GHz; this band is observed in the range of 13.655 GHz to 14 GHz. Therefore, in this mode, a maximum bandwidth of 2.54 GHz is achieved. 

DS-4 shows the five bands of reflection coefficients with less than −10 dB value. For example, the first band represents an S_11_ of −14.420 dB at 3.1 GHz; this band is observed in the range of 3.065 GHz to 3.215 GHz. The second band represents an S_11_ of −20.308 dB at 3.55 GHz; this is observed in the range of 3.485 GHz to 5.655 GHz. The third band represents an S_11_ of −16.715 dB at 7.6 GHz; this band is observed in the range of 7.2 GHz to 7.97 GHz. The fourth band represents reflection coefficients of −19.805 dB at 11.8 GHz; this band is observed in the range of 9.45 GHz to 11.8 GHz. The fifth band represents reflection coefficients of −13.914 dB at 12.57 GHz; this band is observed in the range of 12.55 GHz to 12.585 GHz. In this mode, a maximum bandwidth of 2.35 GHz is achieved.

Table 3 gives an idea about how each modification affects the performance of structure in terms of reflection coefficient, bandwidth, and isolation. Table 3 shows that adding slots in the ground region enhances the reflection coefficients. Therefore, the engraved patch area with surrounded slots and ground region provides the maximum bandwidth of 2.485 GHz.

**Figure 6:**
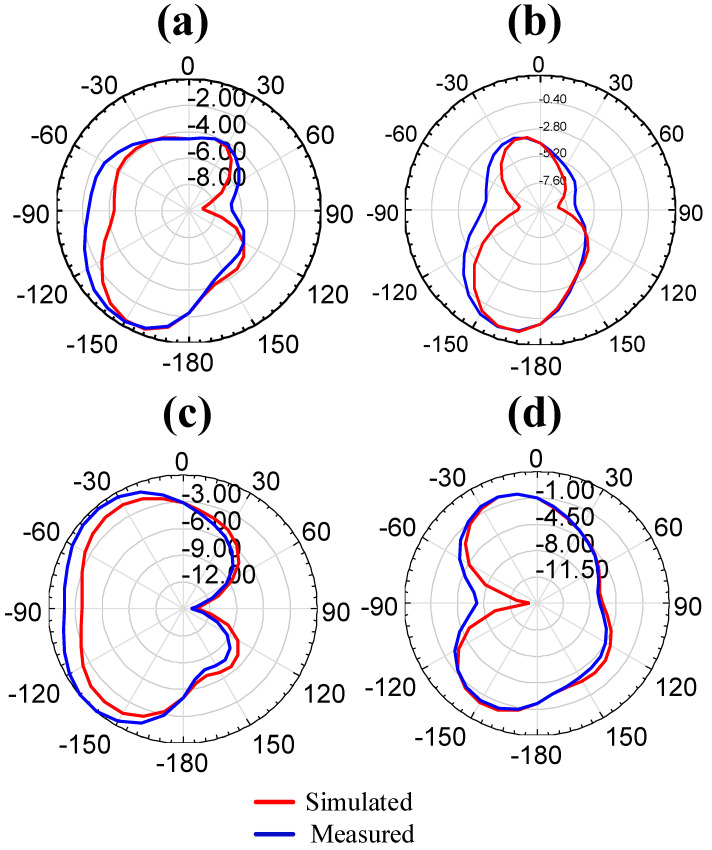
The radiation pattern of four designed structures. (**a**) DS-1. (**b**) DS-2. (**c**) DS-3. (**d**) DS-4. The broadside directivity is observed in DS-3.

The radiation pattern for the simulation and measurement of the proposed antenna structure is represented in Figure 6. The healthy similarity that is observed in both of the results confirms the effectiveness of the structure. The maximum broadside radiation pattern is observed in DS-3. The addition of slots in the patch and ground region affects the radiation pattern. The E-Plane radiation pattern of all of the modes is observed for ϕ = 0°. Practically, antenna gain is measured in the anechoic chamber by keeping the horn antenna [27,28].

**Figure 7:**
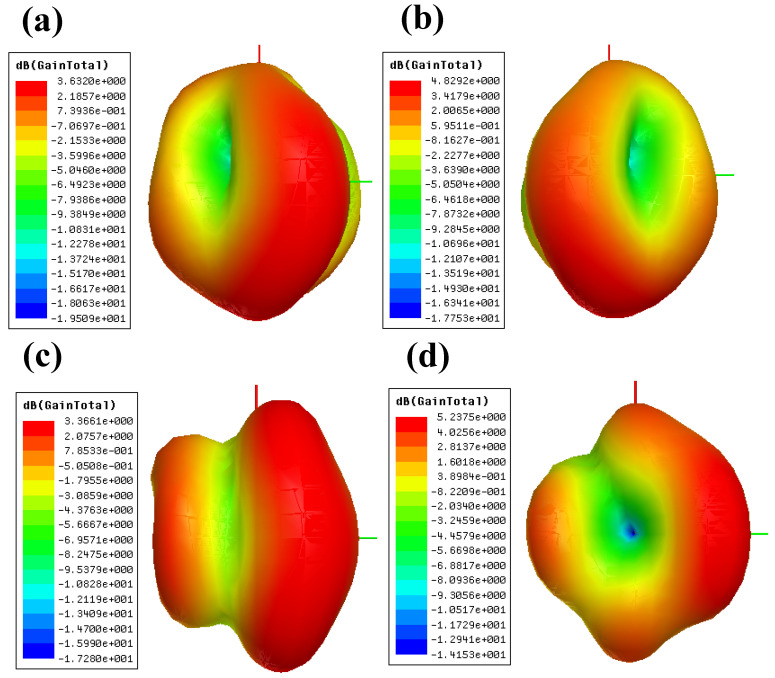
Total gain plot of proposed four MIMO antenna structures. The maximum gain is attained for the DS-4 configurations. (**a**) DS-1 represents total gain of 3.63 dB (**b**) DS-2 represents total gain of 4.82 dB (**c**) DS-3 represents total gain of 3.36 dB (**d**) DS-4 represents total gain of 5.23 dB.

The three-dimensional total gain plot of the proposed MIMO structures is presented in Figure 7. The total gains of DS-1, DS-2, DS-3, and DS-4 are 3.63 dB, 4.82 dB, 3.36 dB, and 5.23 dB, respectively. Maximum gain occurs in D.S.-4 design.

**Figure 8:**
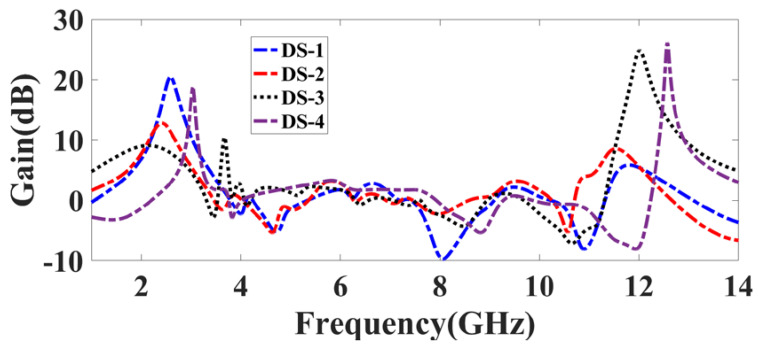
Analysis of peak gain for 1-14 GHz. The peak gains of DS-1, DS-2, DS-3, and DS-4 are, correspondingly, 20.44 dB, 12.79 dB, 24.77 dB, and 26.127 dB.

**Figure 9:**
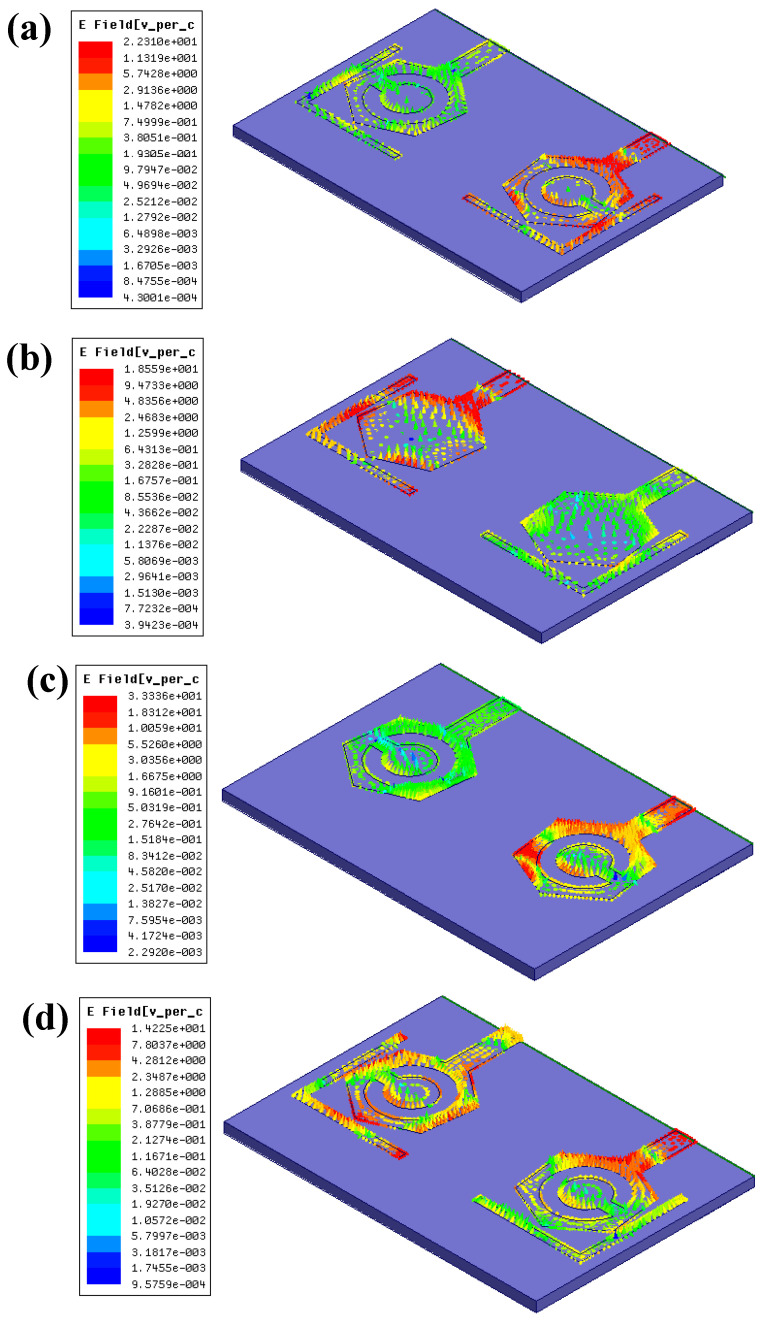
The E-fields of different structures. (**a**) DS-1 represents E-field of 22.3 V/cm (**b**) DS-2 represents E-field of 18.5 V/cm (**c**) DS-3 represents E-field of 33.3 V/cm (**d**) DS-4 represents E-field of 14.2 V/cm.

The gain over the 1 to 14 GHz span is presented in Figure 8. The DS-1 configuration provides an average gain of 1.108 dB attained with a peak gain of 20.44 dB at 2.59 GHz. The DS-2 configuration provides an average gain of 1.42 dB attained with 12.79 dB at 2.415 GHz. The DS-3 configuration provides an average gain of 3.281 dB attained with the peak gain of 24.77 dB at 12.02 GHz. Finally, the DS-4 configuration provides an average gain of 1.16 dB attained with a peak gain of 26.127 dB at 12.575 GHz. The E-field distributions through the patch region for the proposed four designs are shown in Figure 9. The E-fields of DS-1, DS-2, DS-3, and DS-4s are 22.3 V/cm, 18.5 V/cm, 33.3 V/cm, and 14.2 V/cm, respectively. However, due to the opposite field distribution in the slits near the hexagonal-shaped patch area, the effective value of the electric field is reduced [29].

**Figure 10:**
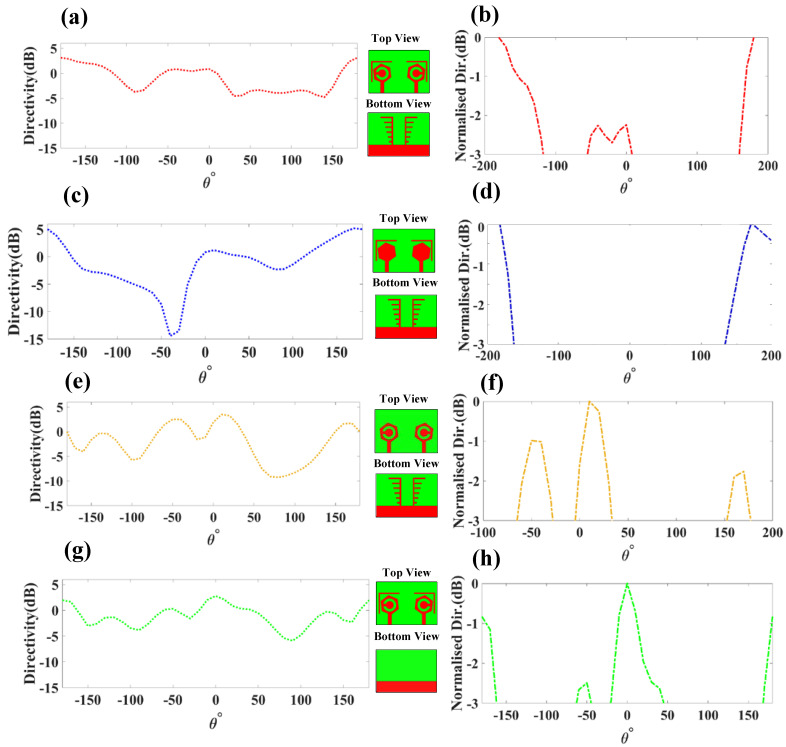
Directivity response for DS-1, DS-2, DS-3, and DS-4. (**a**) DS-1 represents peak directivity of 3.12 dB. (**b**) Normalized directivity of DS-1. (**c**) DS-2 represents peak directivity of 5.16 dB. (**d**) Normalized directivity of DS-2. (**e**) DS-3 represents peak directivity of 3.48 dB. (**f**) Normalized directivity of DS-3. (**g**) DS-4 represents peak directivity of 2.78 dB. (**h**) Normalized directivity of DS-4.

The directivity and −3 dB down directivity plots of the proposed four structures are shown in Figure 10. The co-polar directivity plot is represented over the −180 degrees to +180 degrees range [30]. The peak value of directivity for DS-1 is 3.12 dB. The DS-2 provides a high gain of 5.16 dB. DS-3 provides less directivity than DS-2. DS-3 provides a directivity of 3.48 dB, and DS-4 provides directivity of 2.78 dB. Normalized directivity is calculated at −3 dB down to the peak directivity. The two bands of normalized directivity for DS-1 are 90° and 50°. The normalized directivity for DS-2 is 60°. Three bands of normalized directivity attained for the DS-3 configuration are 40°, 38°, and 30°. Similarly, three bands of 20°, 70°, and 35° are attained for the DS-4 configuration.

**Figure 11:**
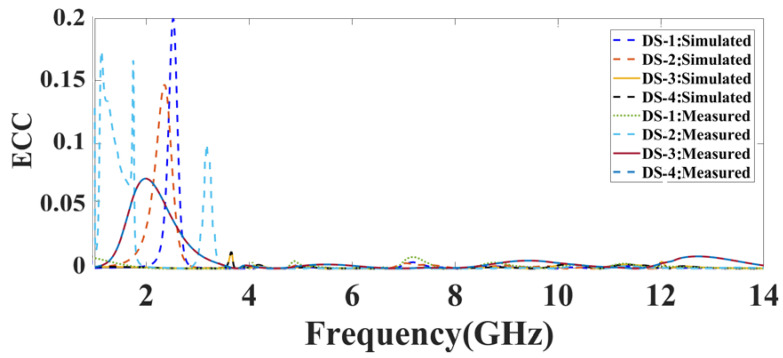
The simulated and measured ECC response of the proposed MIMO antenna structure for the different design structures: DS-1, DS-2, DS-3, and DS-4.

The value of ECC should be zero. The isolation between the individual antenna nodes improves as the ECC increases. Using the scattering parameter, the ECC can be calculated as per Equation (5). The simulated result represents the ECC response of the proposed DS:1, DS:2, DS:3, and DS:4, as represented in Figure 11. DS:1 showed a peak ECC value of 0.2 dB at 2.5 GHz, DS:2 showed a peak ECC value of 0.15 dB at 2.3 GHz, and DS:3 and DS:4 almost showed values of zero for the ECC for the entire spectrum. The measured responses of the ECC for all of the proposed designs, DS:1, DS:2, DS:3, and DS:4, are within an acceptable range or are near to zero over the 1 GHz to 14 GHz range.

(5) $$ECC=\frac{{\left|S_{11}^{*}S_{12}+S_{21}^{*}S_{22}\right|}^{2}}{\left(1-\left({\left|S_{11}\right|}^{2}+{\left|S_{21}\right|}^{2}\right)\right)\left(1-\left({\left|S_{22}\right|}^{2}+{\left|S_{12}\right|}^{2}\right)\right)}$$

The improvement in the SNR of a multiple element system over a one element system is referred to as diversity gain (DG). The DG is calculated using the following Equation (6).

(6) $$DG=10\sqrt{1-|ECC{|}^{2}}$$

**Figure 12:**
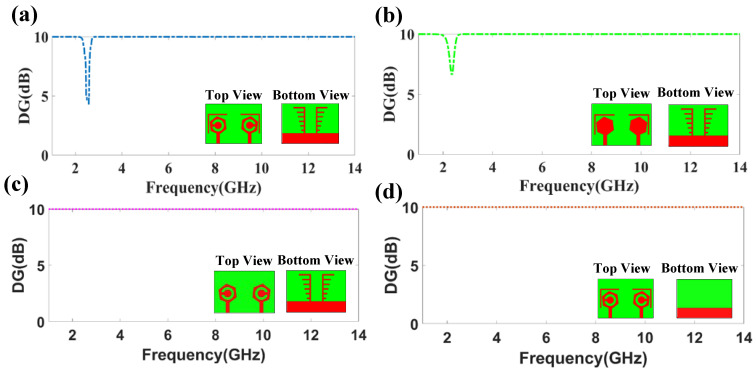
Diversity gain of four proposed design structures. (**a**) DG of DS-1. (**b**) DG of DS-2. (**c**) DG of DS-3. (**d**) DG of DS-4.

The diversity gain of the planned four design structures is illustrated in Figure 12. The DG is analyzed over the 1 GHz to 14 GHz range. The DG value almost remains the same, nearly 10 dB for all of the proposed structures. In DS-1, the DG value reduced by around 4.6 dB at 2.3 GHz. Regarding DS-2, the DG value reduced to nearly 6.8 dB at 2.3 GHz.

The power received by a diversity antenna relative to that received by an isotropic antenna is the definition of a fading environment. The mean effective gain (MEG) is determined by solving Equation (7).

(7) $$\begin{array}{l} MEG1=0.5\left[1-|S11{|}^{2}-|S12{|}^{2}\right]\\ MEG2=0.5\left[1-|S12{|}^{2}-|S22{|}^{2}\right]\\ MEG=MEG1/MEG2 \end{array}$$

A high gain is a need for any respectable MIMO system. The proper MEG response indicates that its diversity performance has been enhanced. Evaluating TARC is the best way to assess radiation performance and frequency response. TARC considers both mutual coupling and accidental signal pairings between ports. The waves that are reflected and incident are used to form Equations (8) and (9), which may be used to obtain the value in terms of the S parameters/

(8) $$\Gamma_{a}^{t}=\frac{\sqrt{\Sigma_{j}^{M}{\left|b_{j}\right|}^{2}}}{\sqrt{\Sigma_{j}^{M}{\left|a_{j}\right|}^{2}}}$$

(9) $$\Gamma_{a}^{t}=\sqrt{\frac{{\left|S_{11}+S_{12}e^{j\theta }\right|}^{2}+{\left|S_{21}+S_{22}e^{j\theta }\right|}^{2}}{2}}$$

**Figure 13:**
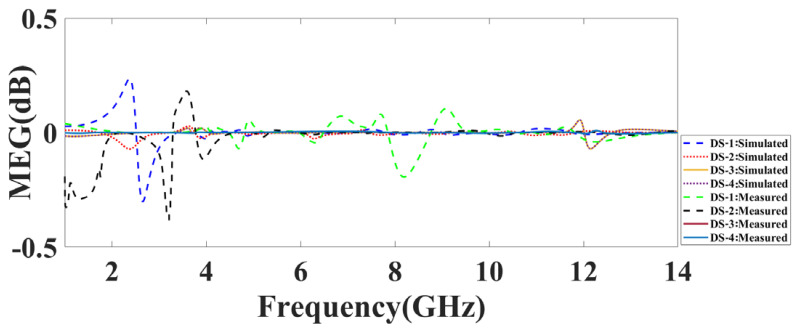
The simulated and measured MEG response of the proposed DS-1, DS-2, DS-3, and DS-4.

The measured and simulated MEG response of the proposed MIMO antenna is represented in Figure 13. It is observed that for all of the proposed design antenna structures, the value is near zero. DS:1 represents the deviation of MEG in the 0.2 to −0.33 range. The MEG response of DS:2 is within the 0.18 to −0.37 range. The representative values of DS-3 and DS-4 are near to zero. The authentication of the MEG response is checked by comparing the simulated and measured response. All of the measured MEG responses of all of the structures are within the permitted range.

In the above equations, the phase of the incident wave is θ. b_j_ and a_j_ stand for reflected and incident waves, respectively. Channel capacity loss (CLL) is another crucial factor to consider while assessing the projected antenna’s MIMO performance. CCL specifies the maximum possible data transfer rate that suffers significant degradation. To prove lossless data transmission, a well implemented MIMO system should achieve a rate of 0.5 bits/s/Hz. Simply said, CCL alerts the user to the point at which further communication cannot be conducted without risk. CCL may be computed using either Equation (10) or Equation (11), respectively.

(10) $$CCL=-{log}_{2}Det\left(\emptyset^{R}\right)$$

(11) $$\begin{array}{l} \emptyset^{R}=\left[\begin{array}{cc} \emptyset_{11} & \emptyset_{12}\\ \emptyset_{21} & \emptyset_{22} \end{array}\right]\\ \emptyset_{11}=1-\left({\left|S_{11}\right|}^{2}+{\left|S_{21}\right|}^{2}\right)\\ \emptyset_{22}=1-\left({\left|S_{22}\right|}^{2}+{\left|S_{12}\right|}^{2}\right)\\ \emptyset_{12}=S_{11}^{*}S_{12}+S_{21}^{*}S_{22}\\ \emptyset_{21}=S_{22}^{*}S_{21}+S_{12}^{*}S_{11} \end{array}$$

**Figure 14:**
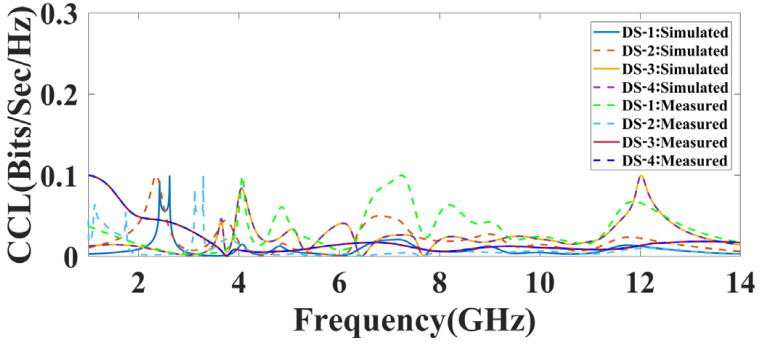
The simulated and measured CCL response of the proposed DS-1, DS-2, DS-3, and DS-4.

Here, the main goal behind these four phases is to achieve a higher gain, which was achieved using the proposed MIMO structures. The simulated and measured channel capacity loss response for all of the proposed design structures is represented in Figure 14. The CCL response, both simulated and measured, is lower than 0.1 for all of the proposed design structures and within an acceptable range.

**Figure 15:**
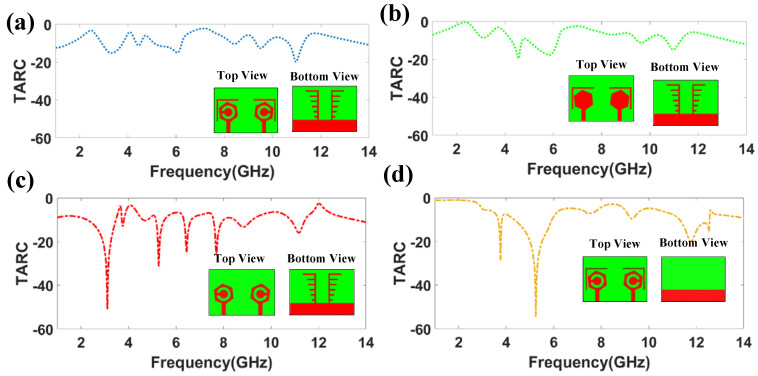
TARC of proposed four MIMO structures. (**a**) TARC of DS-1. (**b**) TARC of DS-2. (**c**) TARC of DS-3. (**d**) TARC of DS-4.

The TARC values for the proposed four MIMO antennas are shown in Figure 15. For all of the configurations, the TARC value is less than 0 dB. DS-1 represents −19.92 dB at 10.9 GHz. DS-2 represents a TARC value of −19.52 dB near 4.55 GHz. In DS-3, a TARC value of −50 dB is achieved near 3.10 GHz. DS-4 shows a TARC value of −54.55 dB at 5.25 GHz. The peak of TARC was observed near the resonating frequency.

The comparison of the presented MIMO structure with the other structures is shown in Table 4 [31,32,33,34,35,36,37]. A comparison of the design is made for five main aspects of the MIMO design. The first aspect is its isolation which needs to be high, and here, in our design, it is 35 dB. The dimension of the proposed antenna is miniaturized in nature and the substrate used is low-cost, which makes it a good pick for MIMO antenna design. The DG of the proposed antenna is also almost 10 dB. The peak gain of the design is around 20.44 dB, which is higher than that of the other designs. The number of ports is two for the proposed research. It is concluded that the presented work provides a remarkably good peak gain, as well as good isolation and DG. Where, NR represents not reported. 

## 4. Conclusions

An FR4, substrate-based, two-port, loaded MIMO antenna has been proposed. Four types of design structures have been analyzed by cropping the patch region, loading the slits near the hexagonal-shaped patch, and adding/removing the slots in the ground area. The proposed design was analyzed in terms of peak gain, DG, reflection coefficient, total gain, electric field, directivity, and E-field pattern. The proposed structure provides an isolation of 35 dB, DG of 10 dB, and peak gain of 20.44 dB. The reflection response of −39.44 dB with the nine band responses having an S_11_ less than −10 dB is attained in DS-3. The maximum electric field of 33.3 V/cm was attained in DS-3. Overall, the addition of slots around the patch area and DGS improves the performance of the antenna. The presented design targeted multiple wireless communication applications for the range of 1 GHz to 14 GHz. Therefore, the presented design structure has potential applications in satellite communication, vehicle speed monitoring, the military, and other 5G/6G applications requiring high data rate transmission.

## Figures and Tables

**Figure 5:**
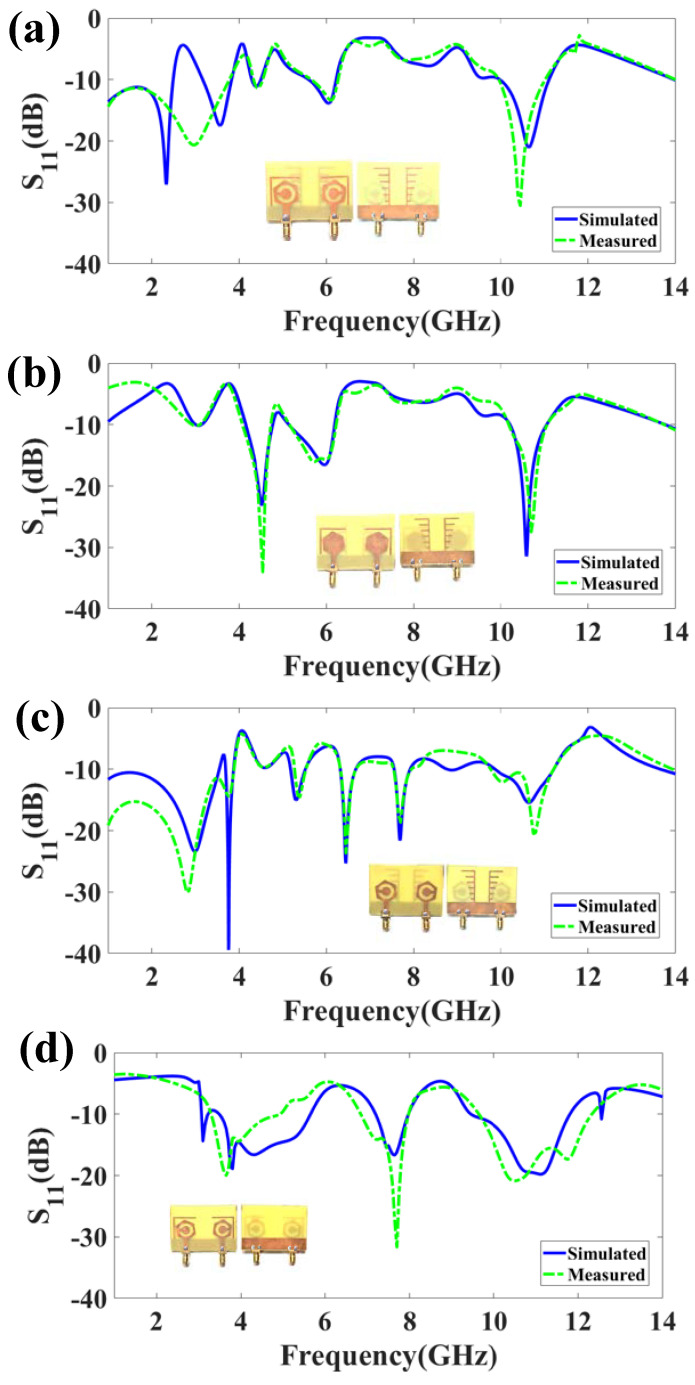
The comparison among measured and simulated results. (**a**) DS-1. (**b**) DS-2. (**c**) DS-3. (**d**) DS-4.

**Table 1:** Four proposed models for the analysis.

Name	Design Structure
Design structure—1 (DS-1)	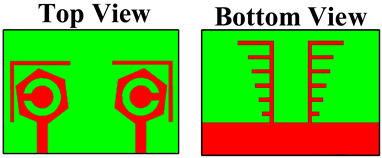
Design structure—2 (DS-2)	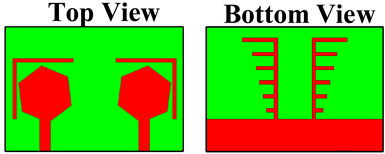
Design structure—3 (DS-3)	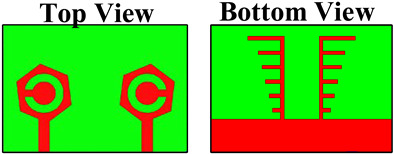
Design structure—4 (DS-4)	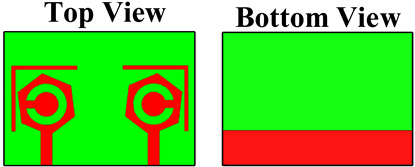

**Table 2:** Different element sizes of the proposed structure. (All units are in mm.)

Parameter	Value
A	1.6
B	2
C	2.3
D	1
E	3
F	3
G	5.4
H	8
I	17
J	17
K	8.4
L	10
M	6.08
N	5.08
O	4.08
P	3.08
Q	2.08
R	9

**Table 3:** Performance comparison among different design structures.

Design Structure	No of S_11_ Bands (<−10 dB)	Maximum Isolation (dB)	Maximum Bandwidth (GHz)	Peak Gain (dB)
D.S.-1	Two	−27.098	2.485	20.44
DS-2	Five	−31.457	1.045	12.79
DS-3	Nine	−39.44	2.54	24.77
DS-4	Five	−20.308	2.35	26.127

**Table 4:** Various MIMO antennas are compared to the presented design.

Reference	Dimension (mm^2^)	Ports	Isolation (dB)	DG (dB)	Gain (dB)
Proposed design	35 × 50	2	35	10	20.44
[31]	30 × 15	2	35.8	>9.99	5.42
[32]	48 × 31	4	21	NR	14
[33]	20 × 20	2	24	9.9	8
[34]	19 × 19	4	35	NR	14
[35]	31 × 31	8	16.1	NR	6.4
[36]	35 × 30	4	17	>9.96	8.3
[37]	12 × 51	4	25	NR	10.6

## Data Availability

The data will be made available upon reasonable request to the corresponding author.
